# Dual-drug loaded nanoneedles with targeting property for efficient cancer therapy

**DOI:** 10.1186/s12951-017-0326-x

**Published:** 2017-12-19

**Authors:** Xiangrui Yang, Shichao Wu, Wanyi Xie, Anran Cheng, Lichao Yang, Zhenqing Hou, Xin Jin

**Affiliations:** 1 Department of Basic Medical Science, Medical College, Xiamen University, Xiamen, 361102 China; 2 Research Center of Biomedical Engineering, College of Materials, Xiamen University, Xiamen, 361005 China; 3 Department of Chemistry, College of Chemistry and Chemical Engineering, Xiamen University, Xiamen, 361005 China

**Keywords:** 10-Hydroxycamptothecine, Methotrexate, Dual-drug, Nanoneedle

## Abstract

**Background:**

Since the anticancer drugs have diverse inhibited mechanisms to the cancer cells, the use of two or more kinds of anticancer agents may achieve excellent therapeutic effects, especially to the drug-resistant tumors.

**Results:**

In this study, we developed a kind of dual drug [methotrexate (MTX) and 10-hydroxycamptothecine (HCPT)] loaded nanoneedles (DDNDs) with pronounced targeting property, high drug loading and prolonged drug release. The anti-solvent precipitation of the HCPT and MTX modified PEG-*b*-PLGA (PEG-*b*-PLGA-MTX, PPMTX) leads to nucleation of nanoneedles with nanocrystalline HCPT as the core wrapped with PPMTX as steric stabilizers. In vitro cell uptake studies showed that the DDNDs revealed an obviously targeting property and entered the HeLa cells easier than the nanoneedles without MTX modification. The cytotoxicity tests illustrated that the DDNDs possessed better killing ability to HeLa cells than the individual drugs or their mixture in the same dose, indicating its good synergistic effect and targeting property. The in vivo studies further confirmed these conclusions.

**Conclusions:**

This approach led to a promising sustained drug delivery system for cancer diagnosis and treatment.

**Electronic supplementary material:**

The online version of this article (10.1186/s12951-017-0326-x) contains supplementary material, which is available to authorized users.

## Background

Duo to the rapid development of the drug resistance in cancer cells [1, 2], the use of a single agent often fails to achieve the all-right therapeutic efficacy. To overcome this problem and improve anticancer efficacy, co-delivery of multifunctional agents is a promising strategy, which have received considerable research interest in cancer therapy [3–5]. It is well known that cancer cells exist at different stages in the cell cycle for the heterogeneity of a tumor and different antitumor drugs have diverse inhibited mechanisms at varying stages of the cell cycle [6, 7]. Thus the delivery system loaded with two or more anticancer drugs would have specific activity on cells at different growth stages and act synergistically. As a result, the combination therapy would bypass the drug resistance of cancer cells and significantly enhance the therapeutic efficiency than individual drug agents [8, 9]. Nevertheless, the combination therapy is largely hindered by their associated side effects, which can deteriorate patient health condition. To address this problem, tumor-specific targeting is proposed for its positive effect on not only reducing the serious side effects, but also enhancing the treatment. Hence, it has become one of the most effective and promising techniques for combination therapy. Folic acid (FA) is one of the most common used targeting ligands, as the folate receptor has been found to be overexpressed on the surface of many types of cancer cells [10, 11]. In recent years, the anticancer drug methotrexate (MTX), whose structure is analogous to that of FA, is also found to have targeting action [12, 13]. Therefore, the MTX loaded in the particles would serve not only as a drug but also as a potential targeting ligand [14]. The targeted therapeutic drug delivery, with MTX as the targeting ligand on the surface cooperated with another anticancer drug inside, was conducive to highly improve the therapeutic efficiency and simplify the nanoparticle-based drug delivery systems simultaneously.

The discussion of dual drug loaded nanostructures could be classified into the nanoparticle-based and carrier-free drug delivery systems. Nanoparticle-based drug delivery systems have received considerable research interest in the past decades [15–19]. As the suboptimal pharmacokinetic properties of the chemotherapy would be significantly improved with the protection of the carrier, such as higher stability, longer circulating half-life, and so on. The nanoparticles that have been demonstrated to deliver therapeutic drugs in combination include polymeric nanoparticles [20–24], polymer–drug conjugates [25–27], mesoporous silica nanoparticles [28], iron oxide nanoparticles [29], and so on. In carrier-based drug delivery systems, the carrier typically make up the bulk of the nanostructures, and the drugs would be loaded in the carrier-based nanostructures via physical adsorption or chemical binding [20–27]. In spite of the improved properties, the low drug loading is the major shortcoming of carrier-based drug delivery systems. On the contrary, carrier-free drug delivery systems have a high drug loading, for the drug make up the major components of the nanostructures [30]. Precisely because of this, the pharmaceutical properties of the carrier-free drug delivery systems may be not as good as those of the carrier-based drug delivery systems. Hence, the concentration of research has been focused on how to combine the advantages of the two systems.

Moreover, another way to improve the efficiency is to change the shape of the nanoparticles. There is already evidence that the shape plays an important role in the cellular internalization, and would affect the result of the treatment to a large degree [31–36]. In our previous studies, it was found that the cancer cells preferred particles with high aspect ratio and sharp ends. The pointed-end, 10-hydroxycamptothecine (HCPT) nanoneedles with an average length of 5 µm were internalized much more rapidly and efficiently by three types of cancer cells than the nanorods with the same size and the nanospheres with a much smaller size of 150 nm [37].

In this study, we developed both methotrexate and 10-hydroxycamptothecine loaded nanoneedles (DDNDs) with high drug loading, targeting and imaging properties. The DDNDs are characteristic of possessing the nanocrystalline HCPT core integrated with the PEG-*b*-PLGA-MTX (PPMTX) conjugated shell, the latter of which functions as the targeting agent and stabilizer as the same time in the system. The nanoneedles with high HCPT loading show the remarkably prolonged and sustained release property due to the presence of the polymeric layer. In the cytotoxicity tests, the nanoneedles showed more excellent killing ability to HeLa cells than the individual drugs or their mixture, which evidenced the good synergistic effect of the dual ingredients and the targeting property of the MTX ingredient. The subsequent in vivo studies further illustrate that the DDNDs has combined the advantages of the carrier-based and carrier-free drug delivery systems. These results highlight the great potential of multidrug-loaded, imaging-functional nanoneedles for highly efficient chemotherapy, as well as for cancer diagnostic applications.

## Methods

### Materials

All the chemicals were analytical grade and used as received without further purification. MTX (purity > 99%) was purchased from Bio Basic Inc. The HCPT (purity > 99%) was purchased from Lishizhen Pharmaceutical Co., Ltd. The monomethoxy (polyethylene glycol)-poly (lactide-co-glycolide) (PEG-*b*-PLGA, PEG: 10%, 2000 Da, PLGA: 20,000 Da, 85/15) was obtained from Daigang Biotechnology Co., Ltd. *N*-hydroxysuccinimide (NHS) and dicyclohexylcarbodiimide (DCC) were purchased from Sigma-Aldrich. The ultrapure water (18 MΩ/cm) was used throughout the work.

### Animals and cell cultures

HeLa cells were was obtained from the American Type Culture Collection. The complete growth medium was DMEM supplemented with 10% FBS and 1% penicillin/streptomycin. The cells were cultivated in an incubator (Thermo Scientific) at 37 °C in the presence of 5% CO_2_ for 24 h.

The BALB/C mice (5–6 weeks, 16–20 g) and BALB/C nude mice (5–6 weeks, 16–20 g) were purchased from Shanghai Laboratory Animal Center, Chinese Academy of Sciences. The tumor models were set up by subcutaneously injecting 1 × 10^6^ HeLa cells in the selected positions of the mice.

### Synthesis of the PPMTX conjugate

MTX (5 mg), PEG-*b*-PLGA (20 mg), DCC (4 mg), NHS (4 mg) and DMAP (2 mg) were added into 2 mL DMF and stirred at rt for 12 h to obtain the PPMTX. Then, the suspension was filtered and dialyzed against a buffer solution (pH 10.0) to remove excess MTX molecules. The remaining suspension was then centrifuged at 5000 rpm and lyophilized for 24 h to obtain the dry PPMTX powder.

### Preparation of DDNDs

First, HCPT (10 mg) and PPMTX (10 mg) were dissolved in 20 mL acetone at 40 °C. Afterwards, the mixture were added dropwise into pure water (100 mL) under sonication (200 W) in an ice bath for 5 min. Then the suspension was centrifuged (10,000 rpm, 5 min) and lyophilized for 24 h to get the DDNDs power. For the preparation of NDs, the PEG-*b*-PLGA was used to replace PPMTX.

### Characterization

Morphology of the DDNDs was examined by SEM (UV-70) at 10 kV. The Size and zeta-potential values were determined by a Malvern Zetasizer Nano-ZS machine (Malvern Instruments, Malvern). Three parallel measurements were carried out to determine the average values. The content of MTX in PPMTX was determined by UV spectrophotometry (Beckman DU800). All samples were assayed at 305 nm. The content of HCPT in DDNDs was determined by fluorescence spectrophotometry (excitation at 382 nm, emission at 525 nm). The content and entrapment efficiency were calculated by Eqs. (1)–(4):

1 $$\begin{aligned} {\text{Drug loading content of HCPT }}({\%}) &= ({\text{weight of HCPT in DDNDs}})/({\text{weight of DDNDs}}) \\ & \quad \times 100{\%}\end{aligned}$$

2 $$\begin{aligned} {\text{Entrapment efficiency of HCPT}}({\%}) &= ( {\text{weight of drug in DDNDs}})/( {\text{weight of feeding drug}}) \\ & \quad \times 100{\%} \end{aligned}$$

3 $$\begin{aligned} {\text{Percentage of MTX in PPMTX }}({\%}) & = ( {\text{weight of MTX}})/( {\text{weight of PPMTX}}) \\ & \quad \times 100{\text{\% }}\end{aligned}$$

4 $$\begin{aligned} {\text{Drug loading content of MTX }}({\%}) &= ( {1 - {\text{Drug loading content of HCPT}}} ) \\ & \quad \times {\text{percentage of MTX in PPMTX}} \times 100{\%} \end{aligned}$$

### In vitro drug release study

The in vitro drug release studies of DDNDs were performed using the dialysis technique. The DDNDs were dispersed in a PBS buffer solution (15 mL) and placed in a pre-swelled dialysis bag (MWCO = 3500 Da). The dialysis bag was then immersed in PBS (0.1 M, 150 mL, pH 7.4 and pH 5.5) and oscillated continuously in a shaker incubator (150 rpm) at 37 °C. All samples were assayed by high performance liquid chromatography (HPLC).

### Confocal imaging of cells

The confocal imaging of cells were performed using a Leica laser scanning confocal microscope with the wavelength of 405 nm as the excitation source. The fluorescent emission was collected from 500 to 600 nm. HeLa cells were incubated in six-well plates at a density of 1 × 10^6^ cells per well. The cells were incubated at 37 °C and 5% CO_2_ for 24 h. The NDs/DDNDs/DDNDs + FA [(HCPT) = 60 µg/mL] were added to the cells for 4 h. After incubation, the cells were washed three times with PBS and fixed with 4% paraformaldehyde. Subsequently, the cells were further washed thrice with PBS before confocal imaging.

### Cellular uptake measured by fluorescence measurement

HeLa cells were seeded in a 24-well plate (5 × 10^6^/well), which was incubated at 37 °C for 24 h in a humidified atmosphere (5% CO_2_). The cells were then incubated with equivalent concentrations of DDNDs/NDs/DDNDs + FA. The drug-treated cells were incubated for 4 h at 37 °C, followed by being washed three times with cold PBS to remove excess nanoparticles. And the cells were then digested with the trypsin (0.05%)/EDTA. The suspensions were centrifuged at 3000 rpm at 4 °C for 5 min. The supernatant was discarded, and the precipitate were washed with PBS to remove the background fluorescence in the medium. After two cycles of centrifugation and washing, cells were resuspended in 2 mL PBS and disrupted by vigorous sonication. The amount of HCPT uptake by cells would release into the sonicated mixture, which was analyzed with fluorescence spectroscopy (excitation at 382 nm). Blank cells without treatment of drug nanocrystals were measured to determine the cells auto-fluorescence level as the control.

### Cytotoxicity assays

The cytotoxicity of DDNDs was determined by MTT assay. Briefly, an adequate number of HeLa cells were planted in quintuplicate in a 96-well plate and incubated for 24 h in the presence of different formulations [(HCPT) = 0.25, 0.50, 1.00, 2.00, 4.00, and 8.00 µg/mL, (MTX) = 0.008, 0.016, 0.032, 0.064, 0.128, 0.256 µg/mL]. In this study, 20 µL 3-(4,5-dimethyl-2-thiazolyl)-2,5-diphenyl-2-*H*-tetrazolium bromide (MTT) solution (5 mg/mL in PBS) was added in each well, and the plate was incubated at 37 °C for another 4 h. Afterwards, a volume of 150 µL dimethylsulfoxide (DMSO) were added, and the plate was agitated in a water bath chader at 37 °C for 30 min. The absorbance at 570 nm was measured using a Microplate Reader (model 680; Bio-Rad).

### Biodistribution

For in vivo fluorescence imaging, DiR was encapsulated into the NDs and DDNDs. DiR-NDs and DiR-DDNDs [(HCPT) = 1 mg/mL] were intravenously administered into the HeLa tumor-bearing nude mice via tail veins at a HCPT-dose of 6 mg/kg. At 1 and 24 h post-injection, the mice were anesthetized and imaged with the Maestro in vivo imaging system (Cambridge Research & Instrumentation, Woburn, MA, USA). After 24 h, the mice were sacrificed, and the tumor and the major organs (liver, kidney, lung, spleen, and heart) were excised, followed by washing the surface with 0.9% NaCl for fluorescence intensity measurement.

In the preparation of DiR-DDNDs, 100 µL DiR was added into the pure water used in the experiment. After sonication, the suspension would dialyze against pure water for 10 h to remove excess DiR. Then the suspension was lyophilized for 24 h to get the DiR-DDNDs power. DiR-NDs was prepared via the same method. Before the biodistribution experiment, several batches of DiR-DDNDs and DiR-NDs would be prepared, and their HCPT drug loading would be characterized. And they would be confected into solutions with the same concentration of HCPT. Then their fluorescence intensity of DiR would be characterized. The solutions whose difference of DiR fluorescence were below 5% would be selected in the experiment.

### Tumor inhibition in vivo

When the tumor volume of the HeLa tumor-bearing mice was approximately 60 mm^3^, the mice were divided into four groups, and treated with 0.9% NaCl aqueous solution, free HCPT and MTX, NDs + free MTX, and DDNDs [(HCPT) = 1 mg/mL] every 3 days at a HCPT-dose of 4 mg/kg per mouse. The tumor volume and body weight were monitored every 3 days. The tumor volume was calculated by the following formula: tumor volume = 0.5 × length × width^2^.

After 21 days, the mice were sacrificed, followed by the tumors excised and weighed. Then, the tumors were fixed in 4% paraformaldehyde overnight at 4 °C, embedded into paraffin, sectioned (4 μm), stained with hematoxylin and eosin (H&E), and observed using a digital microscopy system.

### Statistical analysis

The statistical significance of treatment outcomes was assessed using Student’s t test (two-tailed); P < 0.05 was considered statistically significant in all analyses (95% confidence level).

## Results and discussion

### Synthesis of the PPMTX conjugate

**Fig. 1:**
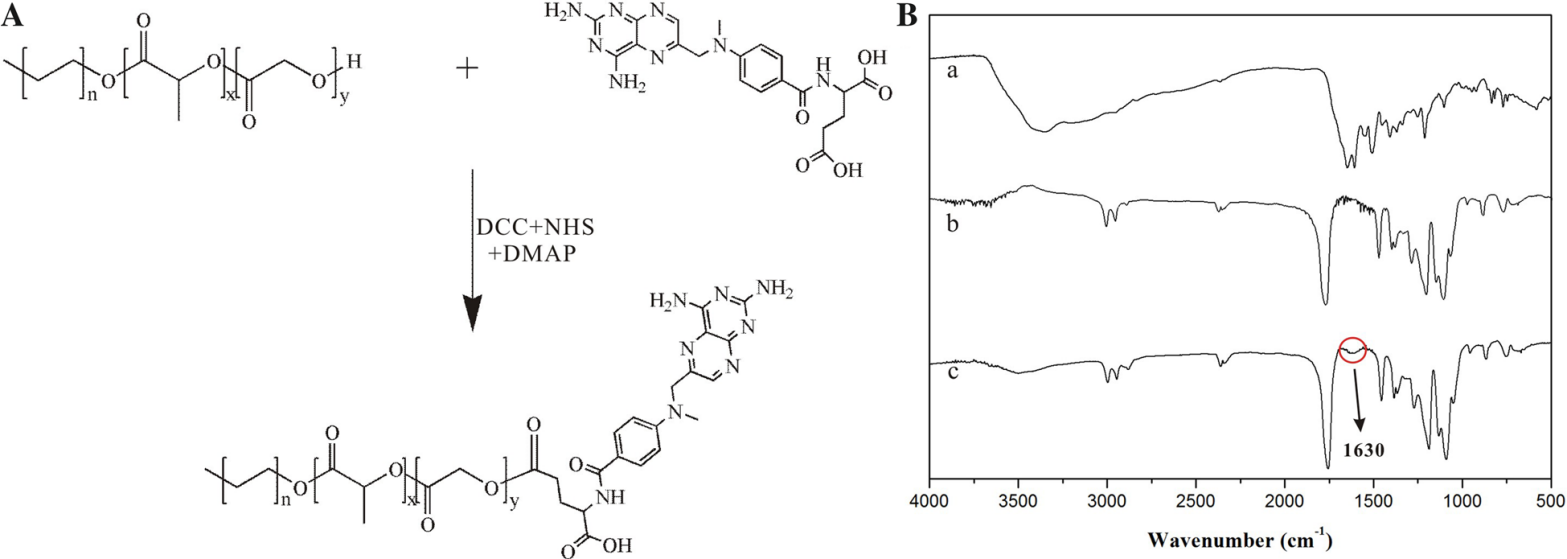
**A** Synthetic route and characterization of PPMTX. **B** FTIR spectra of (a) MTX, (b) PEG-*b*-PLGA and (c) PPMTX

First, we conjugated MTX to PEG-*b*-PLGA by an esterification reaction between the carboxylic end group of MTX and the hydroxy of PEG-*b*-PLGA (Fig. 1A). The structure of the conjugation (PPMTX) was confirmed by Fourier Transform infrared spectroscopy (FT-IR). As shown in Fig. 1B, a new peak at 1630 cm^−1^ appeared in the IR spectrum of PPMTX, corresponding to C=O stretching vibration of the new ester bond. These results indicated that MTX was successfully conjugated to the hydroxy of PEG-*b*-PLGA via ester bond. In order to investigate the percentage of MTX in the conjugation, a standard curve was set up by ultraviolet spectrophotometry. The fitted linear regression equation for the calibration curve was as follows (Additional file 1: Figure S1). And the percentage of MTX was calculated to be 5.1 ± 0.5%.

$${\text{y}} = 0.0474 {\text{x}} - 0.00862 ,\;\;{\text{R}}^{ 2} = 0.9999.$$

(y: the absorbance intensity of UV–Vis; x: the MTX concentration, µg/mL; the detection limits: 1.0–15.0 µg/mL, solvent: DMF).

### Preparation and characterizations of DDNDs

The DDNDs were prepared by an ultrasound-assisted emulsion crystallization method. HCPT and PPMTX were codissolved in acetone, forming a hybrid solution of drug and excipient. When the hybrid solution was injected into deionized water under sonication, a sudden change of solvent environment occurred, inducing the nucleation of HCPT nanocrystallines and the accompanying coprecipitation of PPMTX onto the growing HCPT nanocrystallines [38, 39].

**Fig. 2:**
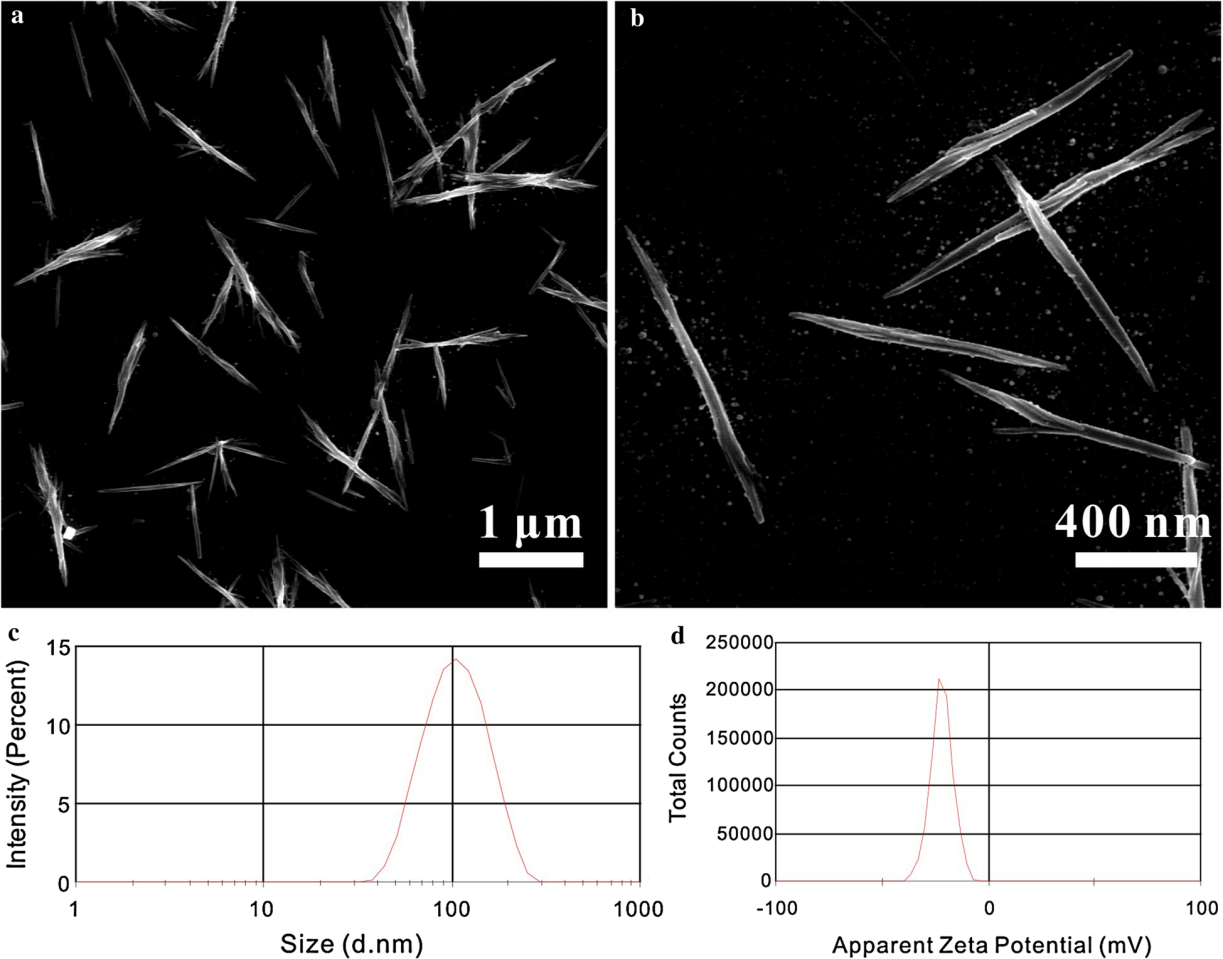
The SEM images (**a**, **b**), the size distributions (**c**), and the Zeta potential (**d**) of DDNDs

Figure 2a, b shows the needle-shaped morphology of the DDNDs with an average length of about 1 μm, and the width of about 80 nm. The result of DLS measurement shows that the DDNDs possessed a size of 102.6 nm (Fig. 2c) and a zeta potential of − 19.3 mv (Fig. 2d). Since only HCPT possessed the property of fluorescent in the drug delivery system, fluorescence spectrophotometry was employed to investigate the drug loading of HCPT in DDNDs. The calibration curve was established (Additional file 1: Figure S2) and the fitted linear regression equation was as below.

$${\text{y}} = 394{,}123{\text{x}} + 10{,}465,\;\;{\text{R}}^{ 2} = 0.99999.$$

(y: the fluorescence intensity; x: the HCPT concentration, µg/mL; the detection limits: 1.0–15.0 µg/mL, solvent: DMF).

It follows that the drug loading content of HCPT was 62.56% and the encapsulation efficiency was 92.43%. And the drug loading content of MTX was calculated to be 2.03%, according to the percentage of MTX in PPMTX.

### In vitro drug release study

**Fig. 3:**
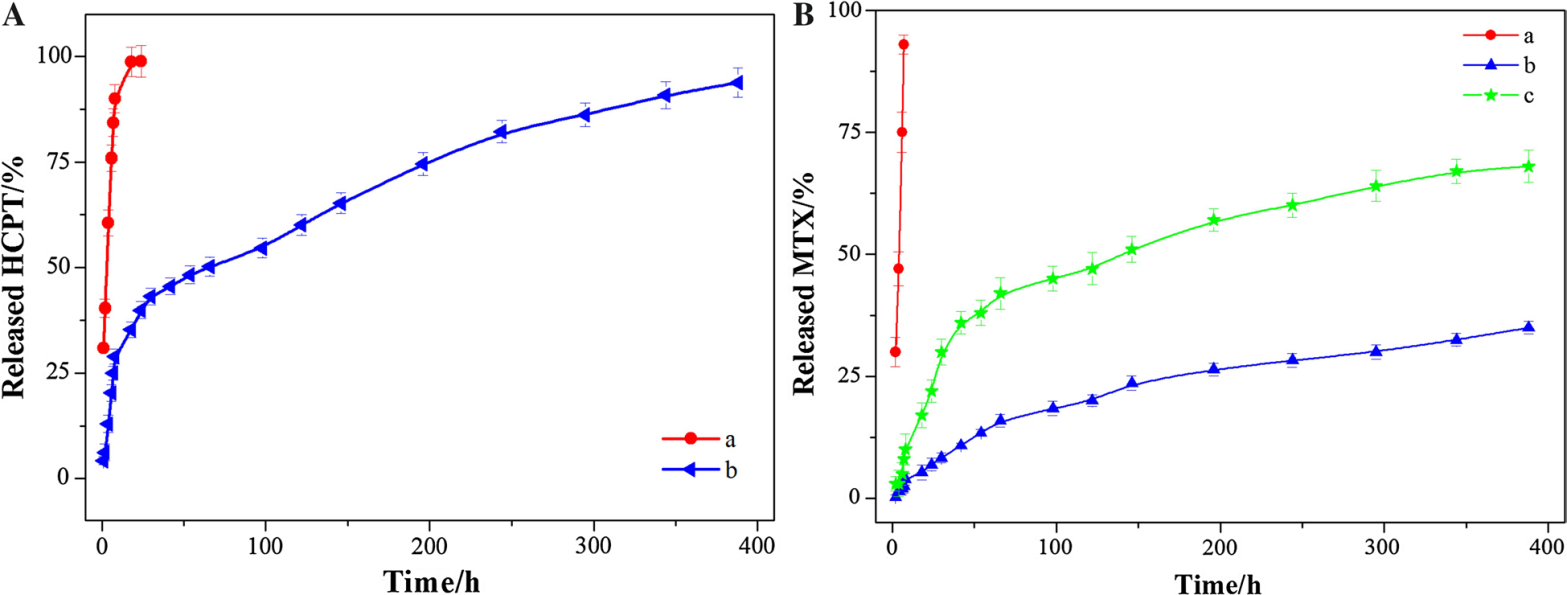
The drug release profiles of free drug and DDNDs under 37 °C and 100 rpm. **A** HCPT: (a) free HCPT (pH 7.4), (b) DDNDs (pH 7.4). **B** MTX: (a) free MTX (pH 7.4), (b) DDNDs (pH 7.4), (c) DDNDs (pH 5.5)

The in vitro release studies of the DDNDs were performed using a dialysis technique, alongside with free HCPT/MTX powders. All samples were assayed by High performance liquid chromatography (HPLC). The release profiles are shown in Fig. 3. The profile of free HCPT powers showed 30% drug-release at the first sampling time of 1 h and nearly 100% by 8 h (Fig. 3A). The HCPT release profile of the DDNDs appears to consist of two components with a slight burst release of about 40% in the first 8 h and followed by a distinctly prolonged release in the next 380 h. This was probably because that the polymeric shell of PPMTX could limit the release of the drug in the core. The profile of free MTX powers was even faster than that of HCPT powers. It only took 4 h to achieve 100% drug-release. However, the MTX release of the DDNDs revealed more remarkably pH-independent and prolonged release, which was most probably attribute to the ester bond between MTX and PEG-*b*-PLGA. Although, a little burst release still existed, the prolonged drug release brought a huge improvement to the free drug. This could compare to the formulations synthesized by other approaches, [39–41] and could greatly promote the application of the DDNDs for sustained drug delivery system.

### Confocal imaging of cells

**Fig. 4:**
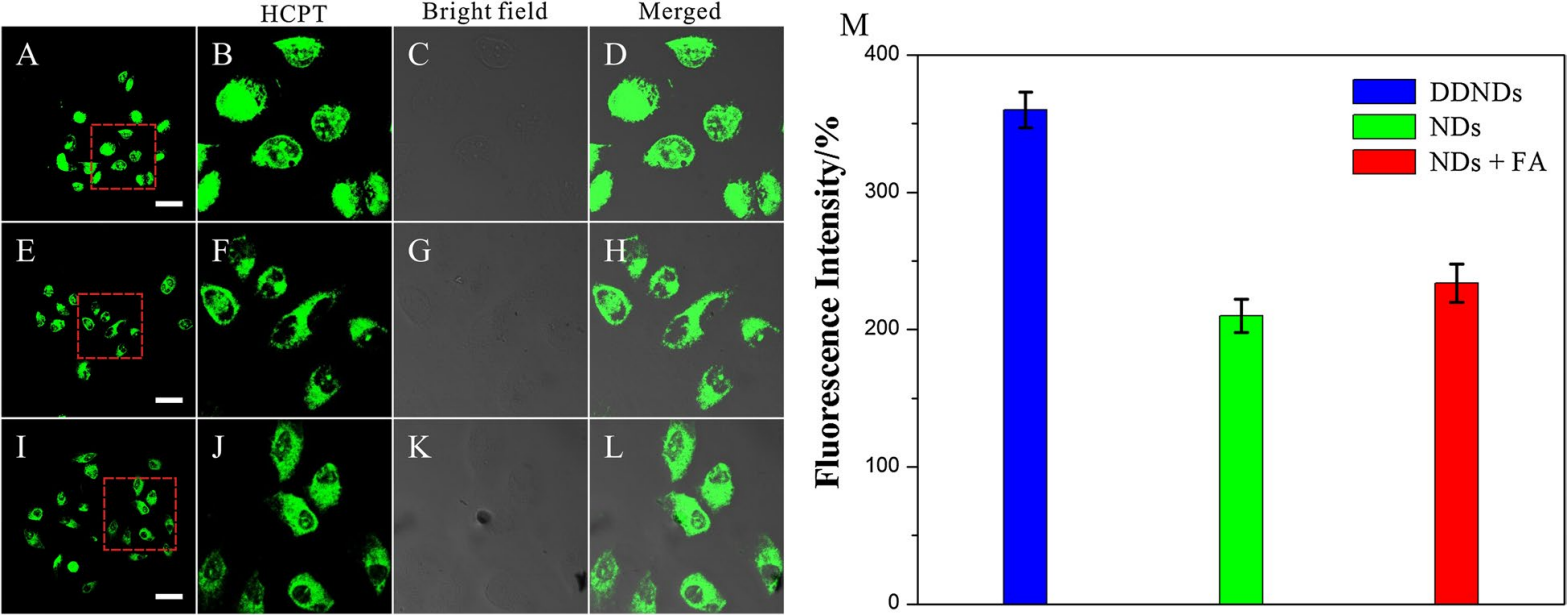
The CLSM images and the fluorescence measurement. The HeLa cells incubated with DDNDs (**A**), NDs (**E**) and DDNDs + folate (**I**) [(HCPT) = 60 µg/mL] for 4 h at 37 °C. All images were taken under identical instrumental conditions and presented at the same intensity scale. All scale bars are 25 μm. **B**–**D**, **F**–**H**, **J**–**L** was the enlarge figure of the red frame in **A**, **E**, **I**, respectively. **M** The fluorescence measurements of the HeLa cells incubated with DDNDs, NDs, and NDs + FA over a 4 h incubation period at 37 °C, P < 0.05

To evaluate their efficiency of cellular uptake by HeLa cells, the DDNDs and the HCPT loaded nanoneedles without MTX modified (NDs, drug loading = 63.6%, d_DLS_ = 121.7 nm) were incubated with HeLa cells for 4 h at 37 °C (The NDs were prepared by HCPT and PEG-*b*-PLGA via the same method as the DDNDs). As shown in Fig. 4A, E, the fluorescence emission of HCPT detected from the cells exposed to the DDNDs was much more intense than that of those exposed to NDs after 4 h of incubation. This illustrated that the MTX on the surface of the particles could greatly enhance the cellular uptake. This was probable duo to its specific affinity to the FA receptors. To further address the specificity of the MTX functionalized nanoparticles for FA receptors, a competition assay was performed. HeLa cells were pretreated with an excess of the free FA (0.50 mg/mL) for 30 min, and then incubated with the DDNDs for 4 h. As shown in Fig. 4I, the fluorescence emissions detected from the group with excess FA molecules became much weaker than that without FA molecules.

When HCPT enter the cells, they would first aggregate in the cytoplasm. This is why the HCPT signals mainly came from the cytoplasm at the group of NDs and NDs + FA (Fig. 4E, I). Nevertheless, there were still weak signals from the nuclei (Fig. 4F, J), which illustrated that HCPT molecules could enter the nuclei with the increase of HCPT concentration. Hence, at extremely high HCPT concentration in the cytoplasm, the nuclei HCPT concentration would increase to a relative high level, which was far more than the limit of detection of CLSM. This led to the result that the HCPT signals from the nuclei were also very intense (Fig. 4A, B), and this phenomena could also been seen in other literatures [42, 43]. Meanwhile, we would still see the difference of the signal intensity between the cytoplasm and the nuclei (Fig. 4B).

The quantification of the fluorescence in the cells also illustrated that DDNDs entered HeLa cells more efficiently than NDs, which would be inhibited by excess FA. This was because the two particles entered the cells via different routes. The NDs were taken into the cells via bulk-phase endocytosis. While the DDNDs can be internalized via the receptors mediated endocytosis as well as the bulk-phase endocytosis. The MTX on the surface of the DDNDs could latch onto FA receptor in the cytomembrane of the HeLa cells and thus enter the cells more efficiently. However, when excess FA molecules were added, they would bind with FA receptors for the enhanced affinities between the FA molecules and the FA receptors. In that case, the DDNDs could not enter the cells via receptors mediated endocytosis, but they could also be uptake via the bulk-phase endocytosis. This was why the Fig. 4C emerged a weak fluorescence.

### Cytotoxicity assays

To further investigate the possibility of utilizing the DDNDs for drug delivery, we tested the killing ability of the DDNDs to cancer cell. The cytotoxicity of DDNDs was evaluated using the MTT assay with the HeLa cells. The NDs, PPMTX, the mixture of NDs and PPMTX containing equivalent concentrations of HCPT or/and MTX were used as control. The concentrations of HCPT were 0.25, 0.50, 1.00, 2.00, 4.00, and 8.00 µg/mL. And the corresponding concentrations of MTX were 0.008, 0.016, 0.032, 0.064, 0.128, 0.256 µg/mL.

**Fig. 5:**
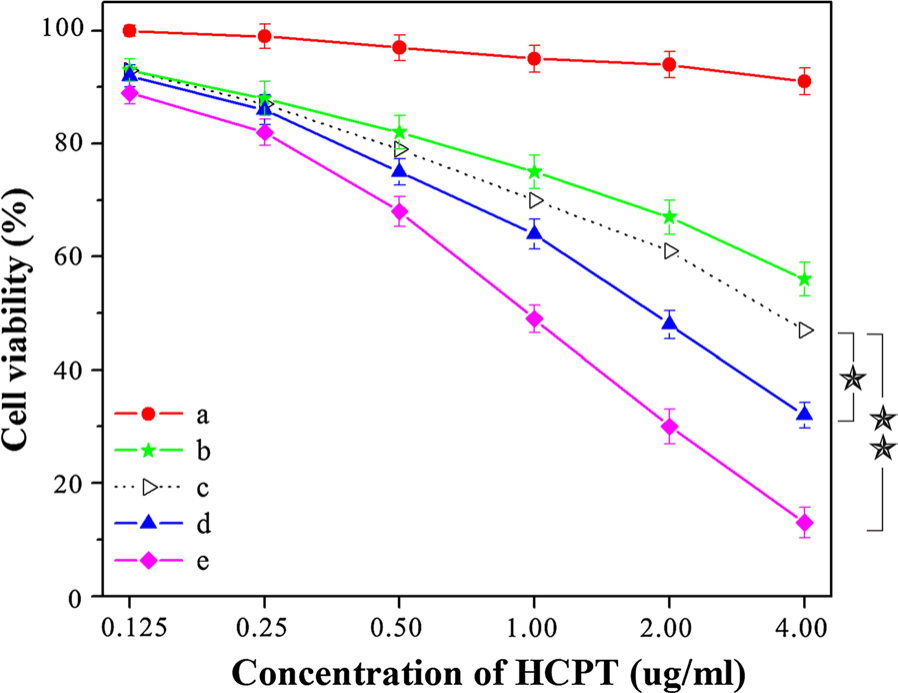
In vitro cell viability of HeLa cells incubated with free MTX (a), NDs (b), NDs + MTX (d) and DDNDs (e) at different concentrations [(HCPT) = 0.125, 0.25, 0.5, 1.0, 2.0, and 4.0 µg/mL; (MTX) = 0.004, 0.008, 0.016, 0.032, 0.064, and 0.128 μg/mL] for 24 h. (c) The theoretical value of free MTX (a) and NDs (b). Data are presented as mean ± SD (n = 6). *P < 0.05

As is shown in Fig. 5a, the PPMTX tend to be nontoxic, mainly because that the concentration of MTX was far below the effective concentration. As to NDs, their cytotoxicity was much higher than that of PPMTX (Fig. 5b. And the theoretical cytotoxicity of the mixture of NDs and PPMTX was calculated by adding the percentage of the cells killed by the NDs and PPMTX. And the experimental cytotoxicity of the mixture of NDs and PPMTX was also tested, which was much higher than the theoretical value. This was because the synergistic effect between the two drugs. MTX could integrate with dihydrofolate reductase to disrupt cellular FA metabolism and then kill cancer cells, while HCPT could inhibit mitosis by acting on DNA topoisomerase I. Hence, the combination of the two drugs would kill the cancer cells through different routes, and act synergistically. Moreover, the cytotoxicity of the DDNDs was even much higher than that of the mixture of NDs and PPMTX. This was probable duo to the targeting property of MTX on the surface of the DDNDs, which could help the particles to enter the cells and kill them. Thus the DDNDs presented surprisingly good killing ability to the cancer cells. This was in according with the result of the CLSM (Fig. 4). These results confirm that MTX on the surface of the DDNDs can increase the cellular uptake of the particles and thus increase their killing ability to cancer cells by binding with FA receptors, just in according with the well-established study [44].

### Biodistribution

To evaluate the tumor target ability of DDNDs, DiR was used as a near-infrared fluorescence probe to be encapsulated into NDs and DDNDs at the equivalent DiR concentration. DiR-NDs, and DiR-DDNDs were injected intravenously into the mice bearing tumors derived from human cervical carcinoma HeLa cells, and their in vivo biodistribution was investigated.

**Fig. 6:**
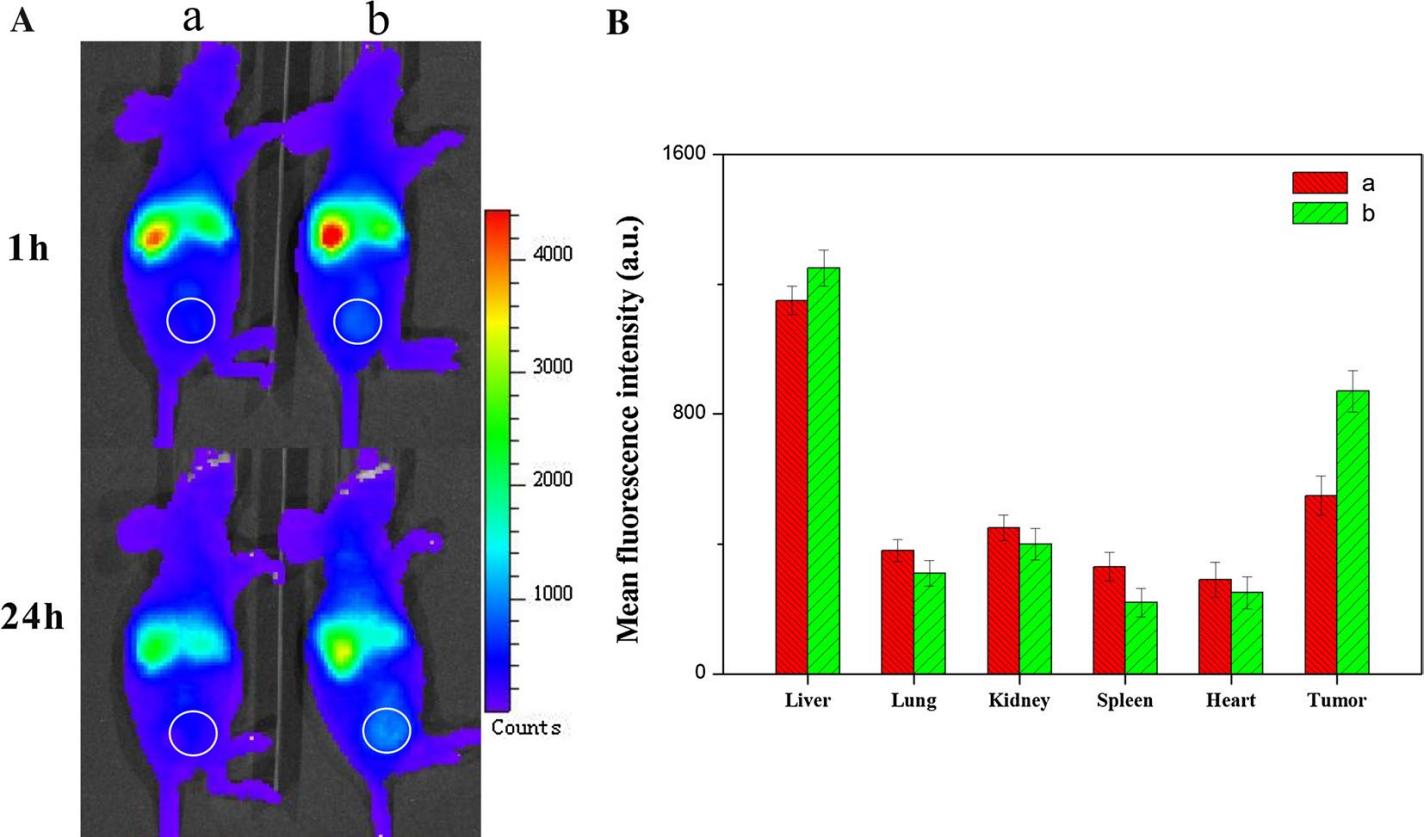
**A** In vivo DiR fluorescence imaging of HeLa tumor-bearing BALB/c nude mice after intravenous injection of the DiR-NDs (a) or DiR-DDNDs (b) at 1.0 and 24 h post-injection. Circles indicated the sites of tumors. **B** Ex vivo fluorescence intensity of tumors and normal organs and tissues harvested from HeLa tumor-bearing Balb/c nude mice intravenously treated with the DiR-NDs (a) or DiR-DDNDs (b) at 24 h post-injection. Data are presented as mean ± SD (n = 3). *P < 0.05

As depicted in Fig. 6A, while no fluorescent signals were detected at tumor sites in the group of DiR-NDs, an obvious fluorescent signal was visualized at the tumor site of the DiR-DDNDs group. When the total fluorescence counts were reduced with the time, the intensity of the signal at the tumor site was enhanced from 1 to 24 h, indicating that the DDNDs were accumulating in tumors during this time. After 24 h, the mice were sacrificed and the tumor tissues as well as the normal tissues were isolated for analysis (Fig. 6B). The fluorescence intensity in the tumor tissue of DiR-DDNDs-treated mice was significantly higher than the other group. It was validated that the introduction of MTX offered the nanoneedles an excellent tumor targeting efficacy, leading to a higher highly efficient cancer treatment.

### Tumor inhibition in vivo

**Fig. 7:**
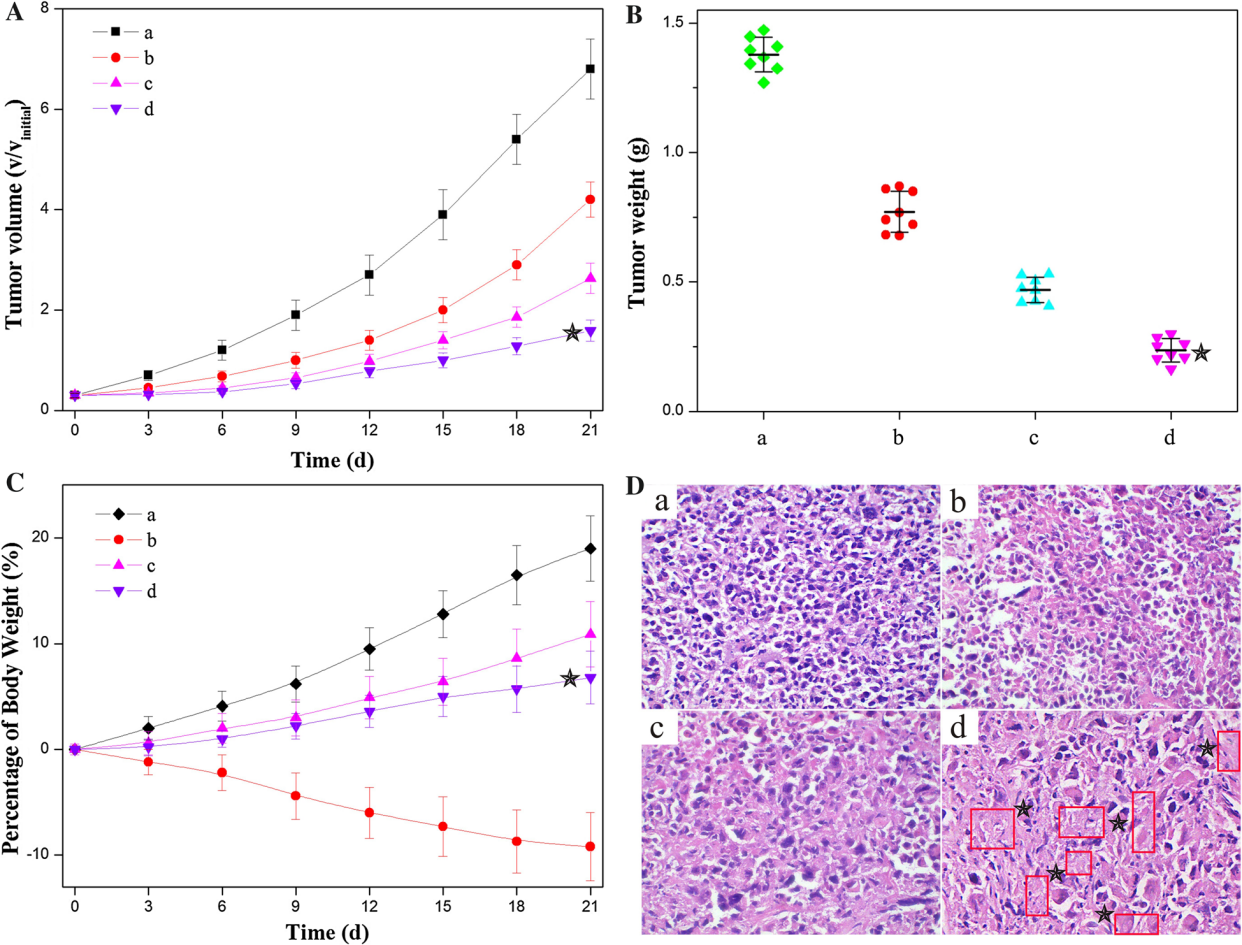
Anticancer effects of different formulations. **A** Volume change of tumor in mice during the treatment. **B** Weights of HeLa tumors after being treated by different (nano)formulations. **C** Weight change of the tumor-bearing mice during the treatment. **D** Histological section of the tumor of the mice after the treatment. (a) 0.9% NaCl aqueous solution, (b) free HCPT and MTX, (c) NDs + free MTX, and (d) DDNDs. All HCPT-MTX formulations used the same concentration of HCPT and MTX in mice bearing HeLa tumor. *P < 0.05

To evaluate the in vivo antitumor effects, we generated HeLa tumor xenografts in Kunming mice and assessed tumor growth following the intravenous administration of 0.9% NaCl, free HCPT + MTX, NDs + free MTX, and DDNDs with the same concentration of HCPT and MTX. Compared to the mice treated with 0.9% NaCl as control, the growth rate of the tumors in mice receiving free HCPT + MTX or NDs + MTX decreased gradually (Fig. 7A, B), indicating the significantly effective tumor growth inhibition. Of note, the DDNDs led to the most pronounced inhibition of tumor growth. At the end of experiment, the tumors were excised and weighed. As shown in Fig. 7C, it was found that the DDNDs had superior therapeutic efficacy compared with the other groups (P < 0.05). An additional evidence of the enhanced anticancer effect of the DDNDs was shown in the histologic images (Fig. 7D). Compared to the control group, several observed necrotic regions could be observed in the tumor section of the group of free MTX and HCPT. More notably, the group of DDNDs displayed the majority of necrosis, indicating their more outstanding anticancer efficacy than other groups. The result suggested that the DDNDs were significantly more effective in inducing cell death and reducing cell proliferation than the combination of the individual drugs or the group of NDs + MTX. This may be owing to the synergistic effect of the two drugs and the targeting effect of MTX on the surface of DDNDs. For any drug delivery systems, the systemic toxicity that is usually encountered in the free HCPT-mediated treatment should be considered to ensure safety and effectiveness. In this work, the administration of the free HCPT + MTX resulted in the listlessness/laziness and severe body weight loss of mice (Fig. 7C), indicative of the undesirable side effects of chemotherapy. On the contrary, no obvious side effects were shown in the mice treated with the DDNDs. Overall, it was indicated that the dual-drug nanoneedles with the superior anticancer effects as well as lower toxicity would greatly improve the efficacy of quality of life therapy.

## Conclusions

The study herein prepared a kind of both MTX and HCPT loaded nanoneedles for the high efficient combination chemotherapy with high drug loading, targeting property and imaging capability. The in vitro drug release profile revealed that the DDNDs showed a sustained and prolonged release. The CLSM images demonstrated the more efficient cellular internalization of DDNDs than that of NDs. The MTT experiment indicated that the DDNDs showed a much higher cytotoxicity than the individual drugs, which illustrated the good synergistic effect of the dual drug. This work opens a door to design new dosages of dual drug loaded nanoparticles.

## Additional file

**Additional file 1: Figure S1.** Standard curves of MTX in DMF via ultraviolet spectroscopy. **Figure S2.** Standard curves of HCPT in DMF via fluorescence spectroscopy.

## Abbreviations

MTX: methotrexate
HCPT: 10-hydroxycamptothecine
PPMTX: PEG-*b*-PLGA-MTX
NDs: HCPT loaded nanoneedles
DDNDs: both MTX and HCPT loaded nanoneedles
