# Supplementary material for: Dual-drug loaded nanoneedles with targeting property for efficient cancer therapy
Source: J Nanobiotechnology. 2017 Dec 19;15:91. doi: 10.1186/s12951-017-0326-x (PMC5735828; doi:10.1186/s12951-017-0326-x)
Supplement: Supplementary file 1 — Additional file 1: Figure S1. Standard curves of MTX in DMF via ultraviolet spectroscopy. Figure S2. Standard curves of HCPT in DMF via fluorescence spectroscopy. [file 12951_2017_326_MOESM1_ESM.docx]

**Electronic Supplementary Information**

**Dual-drug Loaded Nanoneedles with Targeting Property for Efficient Cancer Therapy**

Xiangrui Yang,‡^a,b,c^ Shichao Wu,‡^a,b,c^* Wanyi Xie,^a^ Anran Cheng,^a^ Lichao Yang,^a^ Zhenqing Hou,^b^* and Xin Jin^a^*

^a^Department of Basic Medical Science, Medical College, Xiamen University, Xiamen 361102, China.

E-mail: [wushichao@xmu.edu.cn](mailto:wushichao@xmu.edu.cn). houzhenqing@xmu.edu.cn. xinjin@xmu.edu.cn.

^b^Research Center of Biomedical Engineering, College of Materials, Xiamen University, Xiamen 361005, China.

^c^Department of Chemistry, College of Chemistry and Chemical Engineering, Xiamen University, Xiamen 361005, China.

**The file includes**

Figure S1-2


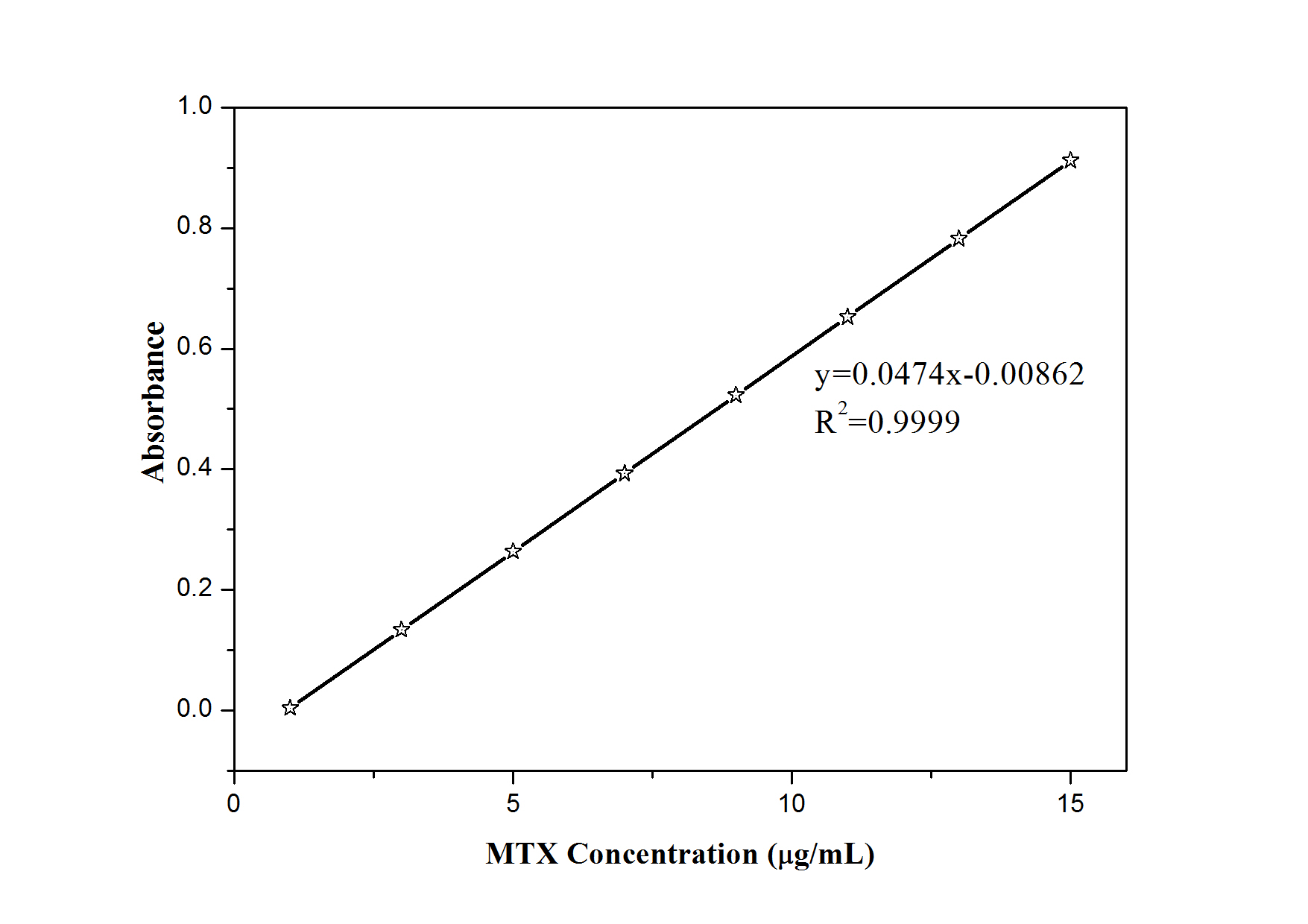


**Figure S1.** Standard curves of MTX in DMF via ultraviolet spectroscopy.


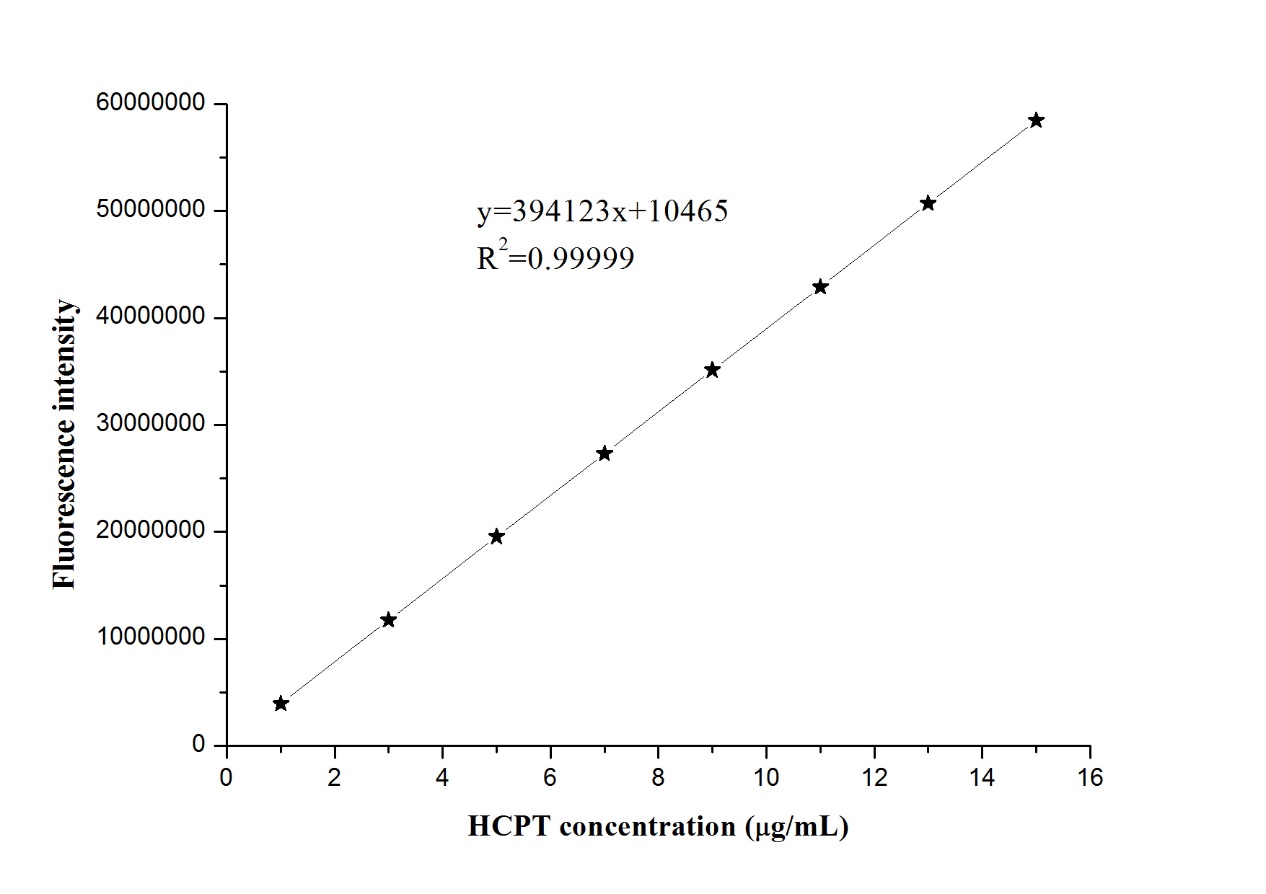


**Figure S2.** Standard curves of HCPT in DMF via Fluorescence spectroscopy.
